# Exploring Factors Associated with Stunting in 6-Month-Old Children: A Population-Based Cohort Study in Sulawesi, Indonesia

**DOI:** 10.3390/nu15153420

**Published:** 2023-08-01

**Authors:** Andi Imam Arundhana Thahir, Mu Li, Andrew Holmes, Adrienne Gordon

**Affiliations:** 1 Central Clinical School, Faculty of Medicine and Health, The University of Sydney, Sydney 2006, Australia; adrienne.gordon@sydney.edu.au; 2 Department of Nutrition, Faculty of Public Health, Hasanuddin University, Makassar 90245, Indonesia; 3 School of Public Health, Faculty of Medicine and Health, The University of Sydney, Sydney 2006, Australia; mu.li@sydney.edu.au; 4 School of Life and Environmental Science, Faculty of Science, The University of Sydney, Sydney 2006, Australia; andrew.holmes@sydney.edu.au; 5 Charles Perkins Centre, Faculty of Medicine and Health, The University of Sydney, Sydney 2006, Australia; 6 The Royal Prince Alfred Hospital (RPA), Newborn Care, Sydney Local Health District, Sydney 2050, Australia

**Keywords:** stunting, poor growth, multiple micronutrient supplementation, infant under 6 months, malnutrition, Indonesia

## Abstract

Stunting in children under the age of two is a significant global concern, particularly in low- and middle-income countries like Indonesia. Intervention efforts often come too late as many of the underlying causal factors have already occurred earlier. While antenatal multiple micronutrient supplements (MMS) have demonstrated positive effects on pregnancy outcomes, their impact on infant growth in the first six months remains inadequately explored in epidemiological studies. This study aims to identify factors associated with stunting at six months in infants whose mothers received MMS. A population-based cohort study was conducted in four subdistricts of Banggai, Indonesia. Pregnant women were recruited in their third trimester and followed up until their children were six months of age. Validated questionnaires were employed to gather data on social determinants of health and diet, and standardised methods were utilised for anthropometric measurements. Stunting was determined based on the WHO child growth standards. The analysis comprised data from 152 mother–child pairs. The prevalence of stunting during early infancy (first two months) was 18.4%, which decreased to 15.8% in later infancy (at six months). Factors such as small-for-gestational-age (AOR = 11.29; 2.73–46.66), preterm birth (AOR = 6.33; 1.25–31.97), short birth length (AOR = 4.31; 1.11–16.78), maternal mid-upper arm circumference (MUAC) below 23.5 cm, and female infants (AOR = 3.27; 95%CI: 1.04–10.27) were associated with stunting at six months. This study highlights that stunting in the first six months is present at birth, with small-for-gestational-age (SGA) as a strong predictor. In addition, there was a trend to improved growth (−0.30 [−0.79 to 0.18]) in infants born to mothers who received MMS supplementation pre-pregnancy rather than during pregnancy, although it was not statistically significant.

## 1. Introduction

Stunting is defined as low length/height for age (length/height-for-age Z-score (LAZ/HAZ) below −2 standard deviations of the WHO child growth standard) that results from chronic or recurrent undernutrition [1]. Stunting is a significant nutritional concern worldwide, especially in low- and middle-income countries (LMICs), including Indonesia [2]. Stunting can lead to numerous negative consequences, including poor cognitive and motor development, morbidities, obesity and associated comorbidities later in life, poor school performance and learning capacity, low productivity, and mortality [3,4,5]. In 2020, the Joint Child Malnutrition Committee of the United Nations Children’s Fund (UNICEF), World Health Organization (WHO), and World Bank (WB) estimated that 149.2 million children under five years old (22.0%) were stunted worldwide; the target of 40% reduction (approximately100 million children) in 2025 is potentially off-track [6].

It is generally accepted that the first 1000 days of life (post-conception) are critical for optimal human growth. Consequently, the nutritional determinants of impaired child growth that characterise stunting may be experienced by the mother, child, or both across this period [3,7]. Epidemiological measures of stunting are necessary to help policymakers assess the incidence, identify contributory factors, and design appropriate interventions. Typically, this is conducted by measuring linear growth retardation with respect to age-specific population-level statistics to serve as markers of poor growth in the population [8]. The age at which this is performed is a potential limiting factor for intervention. For example, epidemiological assessment of stunting at 1 or 2 years of age would likely constrain the capacity to identify associations with potential causal factors that may operate at earlier stages of development and will significantly delay the assessment of public health intervention effectiveness. If stunting is diagnosed at later stages, clinical and public health interventions may also be both harder to implement (owing to the greater independence of the child) and less effective (owing to completed developmental stages).

The incidence of stunting during infancy is an important predictor of both stunting prevalence and cognitive performance in early and late childhood. A previous longitudinal study conducted in the Philippines (*n* = 2131) demonstrated children who first experienced stunting at 1–6 months of age had lower height-for-age Z-scores (HAZ) at 2 years old and 8 years old compared to those who experienced stunting between 7 and 12 months of age (−3.44 vs. −3.02, −2.77 vs. −2.49, respectively). The study also showed that these children tended to have lower cognitive test scores at 8 years old than non-stunted children (−0.35 [−0.48, −0.23]). Therefore, earlier intervention and prevention of stunting is crucial to mitigate its long-term effects and promote optimal growth and development [9]. The development of public health policies focusing on early intervention requires the capacity for early detection. However, there is limited evidence on stunting within the first 6 months of life, with most studies focusing on children aged 6–23 months [10,11,12]. The 6-month time point is an important milestone in an infant’s growth and development, with implications for the infant’s growth trajectory [13]. It is also common when weaning from breast milk to solids occurs. The transition from high dependence on maternal sources of nutrition to dependence on environmental foods is also potentially a shift in the relative importance of causal factors in stunting and, thereby, intervention targets.

Causes of stunting are inherently multifactorial and complex, involving biological, environmental, and socioeconomic factors [14]. Maternal nutrition during pregnancy obviously plays an important role in supporting fetal growth and development [15]. Maternal nutrition is also important postnatally through its influence on the composition and supply of breast milk. However, limited research has evaluated the association between maternal diet during pregnancy and early infant growth trajectory, with most studies focused on birth outcomes [16,17]. There is a need to examine how maternal factors, including diet, nutrition and health during pregnancy, correlate to metrics of stunting across early postnatal life.

In 2018, the Indonesian Basic Health Research survey reported that the prevalence of stunting in children under 2 years across the nation was 30.8%, with a range of 17.7% to 42.6% across different areas [18]. Data on the prevalence and determinants of stunting in children less than six months are currently unavailable. This survey also showed that Banggai Regency is one of the regencies with a high proportion of stunting under two years (31.8%). To address this issue, the government of Banggai introduced a nutritional intervention using multiple micronutrient supplements (MMS) in 2018, replacing iron-folic acid (IFA) supplements. The MMS aimed to bridge the gap in micronutrient intake caused by increased requirements and typically low intake of these essential nutrients observed in LMICs [19]. MMS interventions have the potential to improve pregnancy outcomes, such as birth weight and gestational age, which strongly correlate with stunting [20]. However, until now, there is no available report on the impact of MMS on infant growth and stunting in the first six months of life.

Understanding the causes and timing of stunting is crucial, as stunted children in the early months of life are at a higher risk for (if not more severe growth impairments) stunting in later months. This study aims to explore the factors associated with stunting in children within the first six months in Banggai, Indonesia. Additionally, this study seeks to compare the growth measures of infants in different supplementation groups.

## 2. Materials and Methods

### 2.1. Study Setting and Design

This study was conducted in Banggai, Central Sulawesi Province, Indonesia, from February 2020 to May 2021. The study design was a population-based prospective cohort study that followed pregnant women recruited in the third trimester until six months postpartum. We selected four subdistricts to represent the population: the regency’s capital (Luwuk), sub-districts closer to the regency’s capital (North Luwuk and South Luwuk), and sub-districts far from the regency’s capital (Nambo). This study took place in a setting where childbearing-age women received a multiple micronutrient supplement program from the Banggai government, introduced in 2018 to replace the IFA program.

### 2.2. Study Participant and Sampling

The study participants were pregnant women and their subsequent newborns living in one of the selected sub-districts. Inclusion criteria were 18–49 years of age, 28–36 weeks of gestation, and willingness to remain in Banggai for the next six months postpartum. The exclusion criteria for mothers were as follows: aged 50 years and above and unwilling to stay at the study location after giving birth. There were no specific exclusion criteria for infants, as all infants born to mothers enrolled in this study were included, except if the family moved outside of the study location, or withdrew from study participation. We obtained pregnant women’s information, including their names, addresses, and gestational ages, from the Community Health Centres (Puskemas) or dedicated midwives working in the selected villages. 

After obtaining the agreement to participate, we categorised pregnant women into three groups based on their supplement status. The first group consisted of pregnant women who had received MMS since preconception and continued taking it during pregnancy, called the preconception MMS (PG) group. The second group comprised pregnant women who only received MMS during pregnancy, called the antenatal MMS (AG) group. Finally, the third group consisted of pregnant women who received iron–folic acid supplement (IG) during pregnancy, referred to as the IFA group. It is worth noting that the proportion of this group was relatively small, consisting of only 12 pregnant women (7.9%), as the MMS program has been extensively implemented in Banggai, and IFA supplementation is not commonly consumed. 

We recruited study participants at the integrated service posts (Posyandu) and/or village health posts (Poskesdes). However, due to the COVID-19 pandemic, Posyandu activities were halted, and we had to modify our recruitment procedure. Therefore, we had to recruit participants directly from their homes. First, we contacted the mothers via phone, explained the study’s purpose, and obtained their agreement to participate. We then scheduled a convenient time for the interview and measurement. In addition to direct phone contact, we joined midwives during their home visit program to identify eligible pregnant women. After the interview, we requested the participants to inform the researcher team about acquaintances, neighbours, or family members they knew were pregnant. We contacted these individuals to ask about their interest and consent to participate. The research team followed appropriate COVID-19 protocols and was equipped with sufficient personal protective equipment (PPE) during home visits.

### 2.3. Data Collection

**Figure 1:**
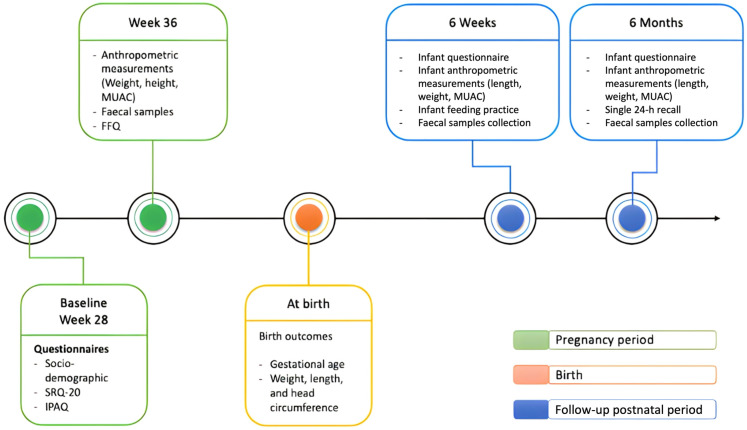
Timeline of data collection, measurements, and follow-up of the study.

This study used open-ended questionnaires adapted from the BABY 1000 study [21]. We used questionnaires in Bahasa Indonesia, which were translated from the first-developed English version. Before data collection, research assistants with a background in health education were trained in data collection and interview techniques. The research team performed interviews after the participants received a brief explanation of the study’s details and then provided signed informed consent. The timeline of data collection and follow-up are presented in Figure 1, with two-time points for maternal data (during pregnancy and at birth) and three-time points for infant data collection (at birth, at six weeks, and at six months). We utilised the anthropometric data obtained at two-time points, TP1 and TP2, for our analysis. Additionally, we examined the mean and standard deviation (SD) of LAZ and the changes in LAZ between the two-time points to reflect the growth differences of the children by supplementation groups.

The anthropometric data included maternal mid-upper arm circumference [MUAC], weight, and height, and child’s MUAC, head circumference [HC], weight, and length. MUAC is an indicator of underweight status for adults [22] and also for pregnant women [23]. MUAC and HC were measured using a SECA 201 measuring tape, and weight, height, and length were measured by using a SECA 203 digital scale, SECA 213 stadiometer, and SECA 210 mat, respectively. We followed the WHO standard procedures to collect anthropometry data, covering recording the date of birth and interview, measuring the child, recording the measurement, and calibrating equipment [24]. 

Maternal nutrition information, including diet and micronutrient supplement consumption, was collected. Mothers’ dietary intake was measured using a validated semiquantitative food frequency questionnaire (FFQ) (Table S1) [25]. Dietary intake during the past three months was measured by selecting the frequency of consuming each food item: per day, per week, per month, and never [26]. Subsequently, responses were converted into average daily intake by considering the serving size. We also measured micronutrient supplement consumption using compliance record sheets. Infant dietary information at six months was collected by interviewing mothers using a 24 h recall questionnaire.

### 2.4. Outcomes and Predictors of Interest

#### 2.4.1. Primary Outcomes

The primary outcome was the prevalence of stunting, determined by age- and sex-specific LAZ z-scores at the first two months (defined as ‘early infancy’ for this cohort) and six months (‘defined as later infancy’) of age. A child was considered stunted if their LAZ score was below −2 standard deviations of the WHO child growth standard [27]. The Z-scores were calculated using the R ‘igrowup’ package, which was provided by the Canadian Paediatric Endocrine Group website [28].

#### 2.4.2. Secondary Outcomes

The secondary outcomes measured in this study included the proportion of small-for-gestational-age infants, which we defined as those born with a weight below the 10th percentile for gestational age. We calculated the weight percentile using the International Fetal and Newborn Growth Consortium for the 21st Century (INTERGROWTH-21st) [29]. Additionally, we also reported the prevalence of short birth length (SBL) as a secondary outcome in this study. We defined SBL children if their birth length was below 48 cm [30].

#### 2.4.3. Associated Factors

We adopted the framework of associated factors from a previous study conducted in Indonesia that examined the social determinants of stunting in children under the age of five [31]. The factors included infant, maternal, and household predictors defined in Table S2. We also collected information on any illnesses the infants had experienced in the two weeks before the interview date.

### 2.5. Data Management, Sample Size, and Analysis

Maternal and infant dietary data were entered into EpiData v. 3.0.0.1 software for macOS and then exported to Nutrisurvey to calculate nutrient compositions based on the Indonesian food composition database from the Ministry of Health of Indonesia [32]. All data were then exported into STATA version 14 for analysis [33]. 

The present study explored the factors associated with stunting, including the gut microbiome of the mother and infant. At the study’s outset, any effect estimates of gut microbiome association with infant growth were unknown, and therefore, the sample size to determine a minimum effect could not be estimated. We set up a pragmatic target of 100 mother–child pairs as the minimum cohort to be recruited within the 12 months prespecified study time period.

The prevalence rate of stunting was calculated as a percentage. We compared the prevalence of stunting between groups using the Pearson chi-square test. We tested all continuous variables for normality using skewness and kurtosis measures and analysed normally distributed data using the student’s *t*-test. For non-normal distribution, we utilised non-parametric methods, such as the Mann–Whitney U-test and the Wilcoxon signed rank analyses. Frequencies and proportions were presented for ordinal and nominal scale variables, and Pearson chi-square or Fisher’s exact tests were performed when comparing these variables in different groups. Due to the small number of mothers in the IG group, they were included in the descriptive analysis only and excluded from univariate and comparative analyses. Logistic regression analyses were conducted on variables associated with stunting to calculate adjusted odds ratios (AORs). We set *p* < 0.05 to determine statistical significance.

The logistic regression of stunting predictors in early and later infancy is presented in Table S3. We considered variables that are clinically important for stunting development, such as supplement compliance, energy consumption during pregnancy, breastfeeding, and complementary feeding, when selecting predictors for our model. Predictors with a *p*-value less than 0.25 in the bivariate analysis were included in a multivariable logistic regression model to control for confounder effects. Before the analysis, we performed a multicollinearity test to examine the interaction between predictors [34].

### 2.6. Quality Assurance Procedures

We performed quality control components to ensure the quality and validity of the collected data and minimise bias as follows:

1. A dissemination meeting involving stakeholders (research team, midwives, and cadres);
2. Anthropometric measurement training;
3. Study protocol training to standardise data collection and input;
4. Calibration of anthropometric measurement tools;
5. Manual checking of questionnaires for completeness by coordinator staff within three days after the interviews. Incomplete responses were clarified with participants via phone or home visits. The verified data were then pooled into the RedCap electronic data management tool hosted at the University of Sydney [35,36].

## 3. Results

### 3.1. Study Participant

**Figure 2:**
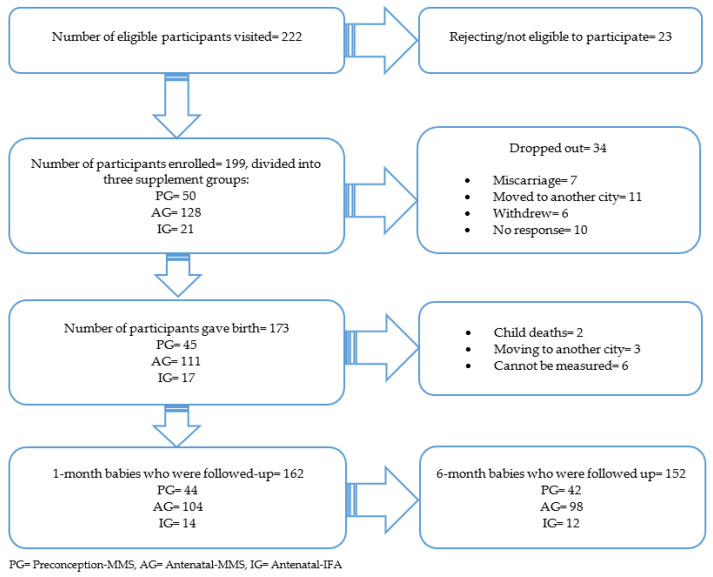
Study participants’ flow chart.

In this study, we approached 222 mothers initially to participate, of which 199 consented to take part. We recruited 104 participants from Posyandu/Poskesdes, while 95 were recruited through home visits during the pandemic. Out of 199 mothers and 173 children enrolled, we finally included 152 mother–infant pairs with a complete growth measurement in the analysis (Figure 2).

The characteristics of the mothers and their infants are presented in Table 1. Of the study participants, 28.3% were primigravida, and 81.6% had minimum ANC visits. Almost 40% of participants came from families with low socioeconomic status, and only 18.4% of the mothers had higher education levels (≥12 years). Most of the participants were living in subdistricts closer to the regency’s capital (44.7%), and only 41.5% were indigenous people (Banggai, Balantak, and Saluan tribes). Of the 152 infants, 44.1% were girls. Approximately 16% of the babies were born small-for-gestational-age, and 25.0% were delivered by Caesarean section.

### 3.2. The Prevalence of Age-Specific Growth Metrics in Supplementation Groups

**Figure 3:**
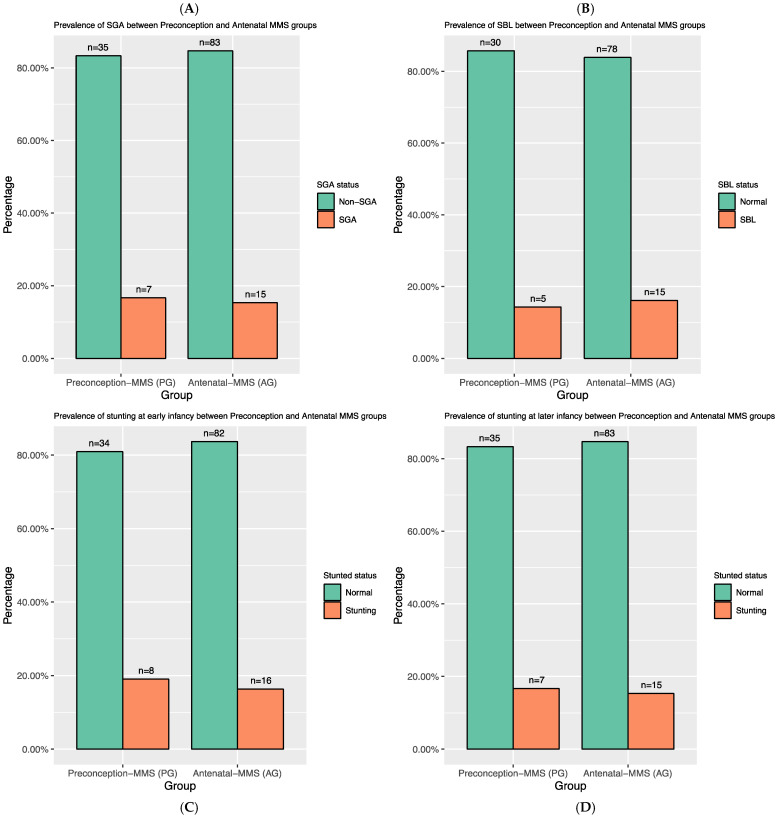
Assessment of prevalence of poor growth at birth (**A**,**B**), at early infancy (**C**) and later infancy (**D**) by supplementation groups (N = 140).

**Figure 4:**
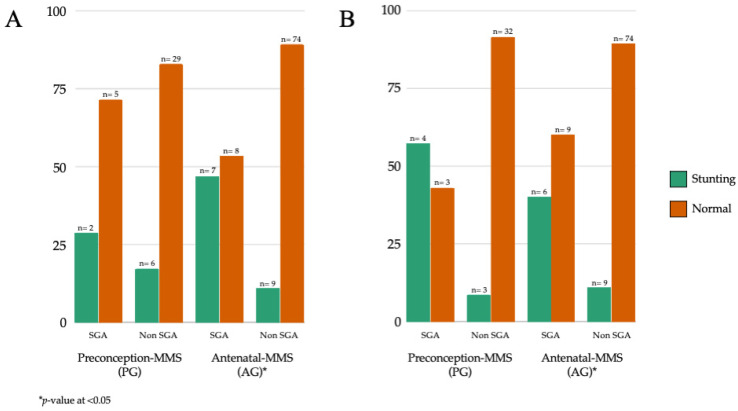
Visualisation of stunting outcomes after maternal MMS provided before pregnancy (PG) and during pregnancy (AG) by size at birth at early (**A**) and later (**B**) infancy (N = 140).

There were no significant differences (*p* > 0.05) in the prevalence of typical stunting metrics between the PG and the AG groups in both early and later infancy (Figure 3C,D). Assessment at birth also found the proportion of SGA and SBL was similar between these two groups (Figure 3A,B). We observed higher stunting in early infancy in the antenatal-IFA group, but the low number of participants in this group meant we were underpowered to test significance. Therefore, we excluded this group from Figure 3 and Figure 4. A finer-grained visualisation displaying the length of boys and girls plotted on the WHO growth chart is shown in Figure S1.

### 3.3. Factors Associated with Stunting in Early and Later Infancy

In this study, the multivariable logistic regression model revealed that size at birth is found to be associated with stunting for both early and later infancy and showed different contributor factors to stunting at different ages, such as infant sex, birth length, preterm birth, and maternal MUAC measures.

In early infancy, this study found that children who were born SGA were 7.67 times more likely to be stunted in the early infancy period than those with normal birth sizes for their gestational age (aOR = 7.67, 95% CI: 1.97–29.91). The odds of stunting in this period were four times higher in female than in male babies (aOR = 4.15, 95% CI: 1.23–13.96). The model also showed that short birth length (aOR = 4.77) and preterm birth (aOR = 6.52) increased the risk of stunting in early infancy (Table 2). Of these factors, the aOR trend was similar, but only SGA (aOR = 7.41) and short birth length (aOR = 5.51) remained significant when assessed at later infancy.

### 3.4. Stratified Analysis of the Association between SGA and Infant Stunting by Supplementation Group

Significant associations were observed between small-for-gestational-age (SGA) and stunting in both early and later infancy among children of mothers from the AG (Antenatal MMS) group (*p* < 0.05). The PG group also showed a significant association of SGA with stunting in later infancy, as shown in Figure 4.

Table 3 presents the difference in infant’s anthropometry and LAZ score between the PG and AG groups. This study found a LAZ difference of −0.23 (95%CI: −0.76–0.30, *p*-value = 0.37) in early infancy and −0.30 (−0.79–0.18, *p* = 0.21) in later infancy between the two groups.

### 3.5. Dietary Intake and Stunting

The intakes of energy and other macronutrients of the mothers and infants are shown in Table 4. The non-parametric Mann–Whitney U test result showed no differences in maternal and infant dietary macronutrient intakes between the stunted and non-stunted groups. In the low socioeconomic group (poor and poorest), dietary fat intake was different between stunted and non-stunted children (*p*= 0.04).

## 4. Discussion

The present study shows that the prevalence of stunting in early infancy (at one month) was 18.4%, which decreased to 15.8% in later infancy (at six months). Our data provide evidence that birth outcomes and maternal nutrition have a substantial effect on stunting prevalence assessed at either period. Specifically, small-for-gestational-age (SGA) was identified as the main factor associated with stunting in early infancy, along with female sex, short birth length, and preterm birth. In later infancy, both SGA and short birth length remained associated with stunting. Additionally, this study found that multiple micronutrient supplements (MMS) administered pre-pregnancy may have a positive effect on infant growth in the first six months compared to those given during pregnancy.

Our findings of stunting prevalence are slightly lower than the 2018 Indonesian Health Research Survey (RISKESDAS), which reported a stunting prevalence of 21.4% among children under six months. It is worth noting that the same survey reported a prevalence of stunting in children under two years at 30.8% [18]. One possible explanation for the higher prevalence among children under two years compared to those under six months is the natural progression of stunting as children grow older, influenced by factors such as prolonged exposure to inadequate environments and nutrition [37]. Although this hypothesis is beyond the aim of our study, it suggests the importance of early intervention in preventing stunting from persisting in older age groups. Some studies have shown that social determinants of stunting are highly varied, ranging from maternal to community-level factors [31,38,39,40]. This variation in predicted determinants likely reflects the interaction between the age-specific nature of stunting assessment with different exposures to factors that may play a causal role in children under and above 6 months of age, most notably diet. Family diet is expected to be correlated to toddler diet owing to shared food availability, but maternal nutrition has the potential to exert a distinct impact on stunting as a mother’s nutritional status can impact both antenatal growth/nutrition of the fetus and the quality and quantity of breast milk she produces [41,42]. While the factors commonly associated with stunting are predominantly postnatal exposures, these are based on growth assessments at 6 months or older [43]. There have been limited assessments of associations with stunting assessed under six months, where we might expect pre- and peri-natal factors impacting maternal nutrition to have a greater impact. 

This study found that poor birth outcomes, such as SGA, preterm birth, and lower birth weight, increased the risk of stunting in children during early infancy. This finding is similar to some published studies conducted in Indonesia, Gabon, Ethiopia, and other developing countries [11,39,44,45,46,47]. The association between poor birth outcomes and stunting may be attributed to the fact that birth outcomes reflect fetal growth conditions, which can affect neonatal health and development [48]. Similarly, SGA has also been associated with stunting assessed at later infancy. Our finding is consistent with a study in the Philippines [49], which reported that SGA was a risk factor for stunting in the first year of life, and SGA infants did not catch up with the linear growth of non-SGA infants from birth to 12 months. Although these studies identify SGA as a predictor of stunting of children in the first six months, it is not commonly used as a measure or health indicator for newborns in Indonesia, especially in Puskesmas (community health centres) in rural areas.

In addition to SGA, this study found that short birth length in newborns (less than 48 cm) was associated with stunting in the first six months of life. Most studies have reported that short birth length is one of the important determinants of stunting [11,38]. One study conducted in Pakistan revealed that various maternal factors, including maternal MUAC, determined normal birth length [50]. A MUAC measurement of less than 23.5 cm is often used to signal protein–energy malnutrition or starvation, which can increase the risk of having LBW infants [51]. It is suggested that maintaining adequate nutrition during pregnancy is essential to promote sufficient growth and maturity of the fetus in the uterus, which in turn helps prevent short birth length and stunting in newborns. In many developing countries, malnutrition is a prevailing challenge even before conception [52]. Therefore, implementing preconception nutritional interventions can be beneficial in preparing for pregnancy and optimising health outcomes for both mothers and children [53].

This study explored the association between preconception nutritional intervention and infant growth during the first six months of age. The findings revealed that although there was no statistically significant difference in length-for-age Z-score (LAZ) between the group that received MMS before conception and during pregnancy, the LAZ trend in both early and later infancy was better in the PG group. This may reflect a potential benefit that might reach statistical significance in a cohort with larger numbers. In addition, it should be noted that both groups consumed MMS on a daily basis. Daily MMS has been shown to reduce the risk of micronutrient deficiencies, prevent maternal anaemia, and improve pregnancy outcomes, especially when given to a healthy population [54]. The WHO currently recommends the use of MMS for women both prior to and during pregnancy [55,56]. Systematic reviews of supplementation comparing MMS with iron–folic acid (IFA) demonstrate the greater effectiveness of MMS in reducing the risk of SGA and preterm birth [20]. In line with this and the current WHO recommendations for MMS supplementation, there were only a small number of women in our study consuming IFA supplements.

Another associated factor with stunting in early infancy was sex, with this study revealing that female babies were 3.3 times more likely to experience stunting than boys. This finding is in line with the results from Nepal, where boys had higher length and LAZ compared to girls throughout the first year of life [57]. In contrast, a study conducted in Ethiopia reported a higher stunting rate among infant boys compared to females [58]. The different findings may be influenced by specific aspects, such as cultural influences. For example, it has been postulated that in some African cultures, female children have a higher value in the local agriculture system [59]. However, limited data exist regarding whether this cultural perspective translates into significant differences in nutritional treatment provided to boys and girls. It is plausible that our study population also has specific cultural practices that predispose to the differential treatment of male and female infants by their parents. However, we did not specifically investigate the cultural factors, limiting our understanding of why girls were more susceptible to stunting in early infancy. Future work in this area is worth exploring.

Our analysis of FFQs did not reveal an association between maternal or infant diets with stunting, which contrasts with the findings of previous studies in similar settings [60,61,62]. However, we found an association with maternal MUAC, which is an indicator of poor nutrition. With a high prevalence of malnutrition and potential food insecurity, the lower quality and variety of available foods may have adversely affected infants’ nutritional status in our study population [63]. For example, children who had a diverse diet comprising food items from five or more food groups demonstrated a lower likelihood of experiencing stunting compared to those who consumed fewer food groups [60]. Additionally, the association between diets and the nutritional and health status of infants may involve other mechanisms, such as the activity of the gut microbiome, which interacts with diet to influence energy harvesting and nutrition [64]. Previous research suggests that the presence of pathogenic bacteria causing enteropathy, particularly in environments with poor hygiene and sanitation, can contribute to stunting [65,66,67,68]. However, we did not assess diet diversity or investigate the role of the gut microbiome, leaving room for further investigation. Conducting research that explores the interplay between food security, gut microbiota, and stunting can provide a more comprehensive understanding of the relationship between diet and stunting.

One of the key strengths of this study is its prospective study design, which allowed for the collection of comprehensive maternal and child data. This study also involved direct interaction with families and conducting door-to-door visits to minimise attrition, despite the challenges posed by the pandemic. The high participation rate and adherence to all measurement protocols further provided a robust dataset and strengthened the reliability of the findings. However, it is important to acknowledge some limitations when interpreting the results. Firstly, the modest sample size of our study may limit the generalizability of the findings to a broader population. Whilst we collected many known variables associated with stunting in this prospective cohort across multiple regions in an LMIC setting, we were unable to include every potential risk factor. Therefore, there are some factors, especially sociocultural, that may be relevant but were unable to be accounted for in this cohort. Nevertheless, this presents an opportunity for future research to validate and expand upon our findings. Further studies with larger sample sizes and diverse populations would enhance the generalizability and provide a more comprehensive understanding of the topic. Thirdly, the use of a single 24 h recall to collect dietary intake information might introduce potential biases. However, the use of three 24 h recalls could be challenging, especially when travel restriction policies were in place during the pandemic. We implemented strategies, such as employing a food model book, to enhance portion size accuracy and utilising the Indonesian Food Composition Database to calculate the nutrient values.

## 5. Conclusions

This study demonstrates the significant impact of size at birth, particularly small-for-gestational-age (SGA), as the strongest early risk factor for stunting in the first six months of life. This study also showed a greater trend of improved growth in infants born to mothers who received MMS supplementation before pregnancy rather than during pregnancy. Thus, implementing targeted nutritional programs is potentially beneficial for this population, with SGA serving as an effective indicator to assess the effectiveness of MMS intervention.

## Figures and Tables

**Table 1:** Characteristics of the study 152 mother–infant dyads.

Characteristics	*n*	%	Mean	SD	Min–Max
**Mothers**					
Age at enrolment (years)			28.5	5.8	18.0–42.0
Height			152.1	5.71	137.7–170.0
BMI at enrolment (kg/m^2^)			27.8	4.6	18.7–40.0
Complete ANC (≥4 visits)	124	81.6			
First time being pregnant	43	28.3			
Supplementary group					
Preconception MMS (PG)	42	27.6			
Antenatal MMS (AG)	98	64.5			
Iron–folic acid (IG)	12	7.9			
Higher education (>12 years)	28	18.4			
Employed	22	14.5			
Socioeconomic status (low income)	60	39.5			
Improved sanitary facility	140	92.1			
Improved source of drinking water	149	98.0			
Residential location					
Regency’s capital	67	44.1			
Subdistricts closer to the regency’s capital	68	44.7			
Subdistricts far from the regency’s capital	17	11.2			
Ethnicity (indigenous/local tribes)	63	41.5			
**Infants**					
Gestational age at birth (weeks)			39.0	1.5	35.1–41.7
Birth weight (g)			3113.9	439.5	2070.0–4200.0
Birth length (cm)			49.1	1.7	45.0–54.0
Gender (girls)	67	44.1			
Small-for-gestational-age	24	15.8			
Preterm birth	17	12.5			
Birth at home	6	4.0			
Mode of delivery (caesarean-section)	38	25.0			
Early initiation breastfeeding	81	53.3			
Exclusive breastfeeding at six weeks	56	36.8			
Commenced complementary feeding at 6 months	117	91.4			

ANC = antenatal care; SD = standard deviation.

**Table 2:** Multivariate logistic regression model of associated factors for stunting.

Associated Factors ^‡^	aOR	95% CI	*p*
**Model 1: Early infancy (R^2^ = 0.45)**			
Female	4.15	1.23–13.96	0.021 *
Small-for-gestational-age	7.67	1.97–29.91	0.003 *
Short birth length	4.77	1.09–20.90	0.038 *
Preterm birth	6.52	1.25–34.05	0.026 *
Mixed or formula feeding	2.19	0.60–7.94	0.23
Do not have family health insurance	1.22	0.15–9.75	0.84
Low supplement compliance	1.84	0.27–12.76	0.53
Maternal energy intake < RDA	5.17	0.33–79.81	0.24
Maternal MUAC < 23.5 cm	1.93	0.36–10.34	0.44
Subdistricts far from the regency’s capital	1.69	0.27–10.45	0.57
Non-indigenous or immigrant	0.45	0.14–1.48	0.19
**Model 2: Later infancy (R^2^ = 0.37)**			
Had morbidity	1.82	0.40–8.18	0.43
Small-for-gestational-age	7.41	1.50–36.67	0.014 *
Short birth length	5.51	1.11–27.45	0.037 *
Incomplete attendance for 4 ANC visits	0.18	0.01–3.31	0.25
Preterm birth	0.41	0.03–6.22	0.52
Not commenced complementary feeding at 6 months	0.83	0.06–10.69	0.88
Low supplement compliance	1.54	0.13–18.62	0.73
Maternal energy intake < RDA	1.38	0.07–28.76	0.83
Maternal height <145 cm	2.00	0.18–22.33	0.57
Maternal MUAC <23.5 cm	2.57	0.36–18.25	0.34
Subdistricts far from the regency’s capital	3.32	0.48–22.91	0.22
Non-indigenous or immigrant	0.64	0.14–2.99	0.57

aOR = Adjusted odds ratio, CI = Confidence Interval; * *p*-value at <0.05. Comparison categories are in brackets; reference categories are not displayed; ^‡^ There is no multicollinearity in the model with the VIF value of <5.

**Table 3:** Infant’s anthropometry and length-for-age z score (LAZ) in the PG vs. the AG groups.

Characteristics	PG Mean (SD)	AG Mean (SD)	PG vs. AG Diff (95% CI)	
			Unadjusted	Adjusted *
Birth weight, g	3141.50 (424.50)	3104.03 (435.27)	−37.47 (−195.04 to 120.10)	−73.92 (−226.01 to 78.17)
Birth length, cm	48.92 (1.57)	49.22 (1.70)	0.30 (−0.35 to 0.95)	0.30 (−0.36 to 0.96)
LAZ in early infancy	−0.39 (1.64)	−0.63 (1.36)	−0.24 (−0.77 to 0.28)	−0.23 (−0.76 to 0.30)
LAZ in later infancy	−0.56 (1.35)	−0.51 (1.17)	0.05 (−0.40 to 0.50)	−0.30 (−0.79 to 0.18)

PG = Preconception MMS, AG = Antenatal MMS; * Adjusted for maternal age, socioeconomic status, height, neonatal sex, gestational age at delivery, energy consumption, and breastfeeding for LAZ in early infancy (*p* = 0.39) and complementary feeding for LAZ in later infancy (*p* = 0.21).

**Table 4:** Dietary intake of pregnant mothers and infants at six months by stunting at later infancy.

Nutrients	Stunted		Non Stunted		Total	
	Median	25th, 75th	Median	25th, 75th	Median	25th, 75th
**Mothers**						
Energy (kcal)	2124.7	2030.7, 2212.0	2093.5	1703.6, 2210.7	2097.1	1759.8, 2211.3
Protein (g)	64.4	55.1, 75.4	65.9	52.6, 80.4	65.5	54.6, 79.4
Fat (g)	35.1	30.8, 43.3	37.6	27.4, 47.5	37.3	27.9, 47.5
Carbohydrate (g)	376.8	349.4, 398.7	357.7	262.2, 395.9	360.1	273.4, 396.7
Fibre (g)	15.1	11.4, 18.6	13.2	10.2, 16.1	13.4	10.4, 16.3
**Infants**						
Energy (kcal)	654.3	563.4, 723.8	673.3	569.6, 790.9	668.0	567.1, 775.8
Protein (g)	12.5	11.0, 14.9	14.0	11.4, 16.8	13.7	11.4, 16.7
Fat (g)	28.2	24.9, 32.4	31.1	24.7, 37.1	29.9	24.8, 36.7
Carbohydrate (g)	84.3	67.0, 92.1	84.3	73.6, 99.2	84.3	73.2, 98.5
**Infants from low socioeconomic households**						
Energy (kcal)	621.4	538.1, 673.2	711.4	575.3, 777.3	673.2	571.0, 772.7
Protein (g)	11.8	9.3, 13.0	14.2	11.3, 16.9	13.8	11.2, 16.7
Fat (g) *	26.4	24.9, 29.1	32.0	26.3, 38.3	29.5	26.1, 36.7
Carbohydrate (g)	81.1	65.9, 84.3	84.0	72.7, 96.8	82.7	70.0, 96.4

* Mann–Whitney U test *p*-value = 0.04.

## Data Availability

Availability of the data will be released upon reasonable request through corresponding authors. The data are not publicly available due to restrictions concerning privacy and ethical reasons.

## Supplementary Materials

The following supporting information can be downloaded at: https://www.mdpi.com/article/10.3390/nu15153420/s1, Table S1: Food Frequency Questionnaires of the Study (FFQ); Table S2: Categories and Definitions of the Study Variables; Table S3: Univariate logistic regression of stunting predictors in early and later infancy; Figure S1: Length of boys (A) and girls (B) plotted on WHO growth chart. References [69,70,71,72] are cited in Supplementary Materials.
