# Supplementary material for: Exploring Factors Associated with Stunting in 6-Month-Old Children: A Population-Based Cohort Study in Sulawesi, Indonesia
Source: Nutrients. 2023 Aug 1;15(15):3420. doi: 10.3390/nu15153420 (PMC10421344; doi:10.3390/nu15153420)
Supplement: Supplementary file 1 [file nutrients-15-03420-s001.zip › nutrients-2497491-supplementary.pdf]

Table S1. Food Frequency Questionnaires of the Study (FFQ).

Respondent ID : ………………………………………………………………

Date : ………/………/………

Coordinator check date : ………/………/………

No	Food group/ type	Weight per portion	Portion	Frequency					
				Never	1 per month	2-3 per month	1-2 per week	3-4 per week	Everyday
**I**	**Carbohydrate**								
	Rice	100	¾ glass						
	Brown rice	100	¾ glass						
	Yellow corn (sweet)	200	1 pc (lrg)						
	White corn	50	½ glass						
	Purple sweet potato	100	1 pc (med)						
	Red sweet potato	150	1 pc (med)						
	Cassava	100	1 pc (med)						
	Rice noodles	100	1 glass						
	Wet noodles	100	1 glass						
	Dry noodles	50	1 glass						
	Instant noodles	68	1 ¼						
	Macaroni	100	1 glass						
	Potato	100	2 pcs (med)						
	Sago powder	40	7 tbsp.						
	Flour	5	1 tbsp.						
	Bread	20	1 slice						
	Wholemeal bread	100	2 slices						
	…..........................								
	…..........................								
	…..........................								
**II**	**Protein**								
	**Animal-source**								
	Beef	50	1 (med) cut						
	Lamb	50	1 (med) cut						
	Meatball	100	10 pcs (lrg)						
	Shrimp	200	1 glass						
	Squid	134	1 pc						
	Mackerel Tuna	50	1 (med) cut						
	Sardine	50	1 (med) cut						
	Skipjack	50	1 (med) cut						
	Dried anchovy	8	1 tbsp.						
	Salted fish	25	1 (med) cut						
	Scallop	9	1 pc						
	Chicken	50	1 (med) cut						
	Shrimp	45	1 (med) cut						
	Liver (Beef)	25	1 (sml) cut						
	Liver (Chicken)	50	2 pcs						
	Duck	45	1 (med) cut						
	Chicken eggs	60	1 pc						
	Free-range chicken eggs	50	2 pcs						
	Quail eggs	55	5 pcs						
	Cheese	15	1 (sml) cut						
	…..........................								
	…..........................								
	…..........................								
	**Plant-source**								
	Tofu	100	1 pc (lrg)						
	Tempe	25	1 (med) cut						

	Soybeans	15	1.5 tbsp.
	Mung beans	25	2.5 tbsp.
	Red beans	25	2.5 tbsp.
	Peeled peanuts	20	2 tbsp.
	…...........................		
	…...........................		
	…...........................		
**III**	**Vegetables**		
	Spinach	125	1 bunch
	Water spinach	100	1 glass
	Mushroom	100	1 glass
	Melinjo/ Ganemo leaves	50	1 glass
	Beans	100	15 pcs
	Sprouts	50	1 glass
	Eggplant/ Popoki	100	1 glass
	Japanese Cucumber	100	1 (med) cut
	Mustard greens	41	1 pc
	Cabbage	100	1 (med) cut
	Broccoli	100	1 (med) cut
	Celery	100	5 bunches
	Carrots	100	1 glass
	Cassava leaves	104	1 bunch
	Potato	200	2 pcs
	Katuk leaves	50	1 glass
	Basil leaves	100	35 pck
	Long beans	15	1 pc
	Cucumber	83	1 pc (med)
	Bitter melon	70	1 pc (med)
	Tomato	43	1 pc (lrg)
	Lettuce	50	1 glass
	Young jackfruit	100	1 glass
	Banana flower/ heart	50	1 glass
	Papaya leaves	50	1 glass
	…...........................		
	…...........................		
	…...........................		
**IV**	**Fruits**		
	Papaya	100	1 (med) cut
	Plantain	50	1 pc (med)
	Ambon baban	75	1 pc (med)
	Pisang mas	40	2 pcs
	Pisang kepok/ pisang sepatu	45	1 pc
	Banana of Ambon	75	1 pc (med)
	Apple	75	1.5 pc (lrg)
	Orange	100	1 pc (med)
	Durian	17,5	1 pc (med)
	Pineapple	73	1 pc (med)
	Water apple	50	1 pc (med)
	Langsat/ Duku	8,9	1 pc (med)
	Kedondong	60	1 pc (med)
	Rambutan	9,4	1 pc
	Mango	50	½ pc
	Melon	150	1 cut (lrg)
	Avocado	50	1.5 pc (med)
	Grapes	75	10 pcs
	Watermelon	150	1 cut (sm)
	Snake fruit	75	1 pc
	Mangosteen	60	1 pc
	…...........................		
	…...........................		

	…...........................		
**V**	**Beverages**		
	Tea	3,7	1 tbsp.
	Yogurt	200	1 glass
	Coffee	182	1 glass
	Cow milk	200	1 glass
	Soya milk	200	1 glass
	Condensed milk	200	1 glass
	Unsweetened condensed milk	200	1 glass
	…...........................		
	…...........................		
	…...........................		
**VI**	**Local foods**		
	Papaya leaves	100	35 pcs
	Ferns leaves	100	25 pcs
	Moringa leaves	100	1.5 glass
	Pisang louwe	50	1 (med) cut
	Pisang kapok	50	1 (med) cut
	Ubi Bete (Sweet potato)		
	Sambiki (Summer squash)	100	1 (med) cut
	Dalundung vegetable	50	1 glass
	Gogos (White glutinous rice)	162	1 pc
	Jaha rice (bamboo-cooked rice)	160	1 pc
	Lalampa (white glutinous rice and fish)	50	1 (med) cut
	Roa (Roa fish)	50	1 (med) cut
	Kadompe (Mackerel)	50	1 (med) cut
	…...........................		
	…...........................		
**VII**	**Seasonings/ ingredients**		
	Margarine	5	½ tbsp.
	Fish oil	5	½ tbsp.
	Palm sugar	8	½ tbsp.
	Sugar	8	1 tbsp.
	Honey	10	1 ¼ tbsp.
	Coconut milk	50	½ glass
	Coconut	30	1 (sml) cut
	…...........................		
	…...........................		
	…...........................		

Table S2. Categories and Definitions of the Study Variables.

Predictors	Categories	Definition
Stunting	Stunted<br>Non-stunted	Height-for-age Z-score (HAZ) below -2 standard deviations less than the median of WHO child growth standard [1].
Gender	Male*<br>Female	Children's sex.
Breastfeeding	Exclusive breastfeeding*<br>Mixed or formula feeding	A way of feeding a baby or type of breastfeeding at six weeks.
Complementary feeding	Commenced complementary feeding at 6 months*<br>Not commenced complementary feeding at 6 months	Refers to the introduction of solids or semi-solids foods into an infant's diet at six months.
Had morbidity during the last 2 weeks	Absence of morbidity*<br>Had morbidity	Children's morbidity history in the last two weeks before the participants' interview.
Small-for-gestational age	Non-SGA*<br>SGA	Newborns size which are smaller according to their gestational age. SGA is defined as weight at birth below the 10th percentile [2].
Birth length status	Normal*<br>Short birth length	Low birth length has been defined as length at birth of <48 cm [3].
Delivery process	Normal*<br>Caesarean section	The method of childbirth delivery of the study participants.
Early initiation of breastfeeding	Early initiation of breastfeeding*<br>Non-early initiation of breastfeeding	Newborn children who were put to the breast within one hour after birth.
Pre-lacteal feeding	No*<br>Yes	Feeding practices in which newborn babies is given with any substance other than breast milk before initiating breastfeeding.
Number of ANC visits	Complete attendance for 4 visits ANC*<br>Incomplete attendance for 4 visits ANC	The number of visits to healthcare provider for receiving antenatal care.
Birth term status	Term birth (37-40 weeks) *<br>Preterm birth (< 37 weeks)	The age of gestation at the time of giving birth. Divided into three categories, term, preterm, and post-term.
Family health insurance	All members have family health insurance<br>Some members have family health insurance<br>Do not have family health insurance	Family members covered by the government (so-called 'BPJS').
Labour at health facility	Yes*<br>No	The location of childbirth delivery of study participant.
Supplementation Group	PG (Preconception-MMS)*<br>AG (Antenatal-MMS)<br>IG (Antenatal iron-folic acid)	Type of supplementation given by the local government of Banggai during the study. Divide into three groups, multi-micronutrient supplementation receiving since pre-pregnancy (PG), multi-micronutrient supplementation receiving during pregnancy (AG), and other supplements receiving during pregnancy (IG).
Supplement consumption compliance	Adequate supplement compliance (>= 90 capsules)*<br>Low supplement compliance (< 90 capsules)	The number of supplements consumed during pregnancy.

Maternal energy intake	Energy intake >= RDA*<br>Energy intake < RDA	Energy intake of the mothers during pregnancy
Maternal height	>= 145 cm*<br>< 145 cm	A maternal height measured at the first visit.
Maternal age at pregnancy	Not at risk (20-35 years old)*<br>At risk (<20 and >35 years old)	Maternal age during pregnancy at first visit.
Mother's education	High (>12 years)*<br>Low (<= 12 years)	Category of the maternal education level.
Mother's employment status	Housewife*<br>Working	Category of the maternal occupation level.
Mental health symptom	None*<br>With symptom	The mental health state of the study participants was measured by self-reporting questionnaires to indicate the presence of mental health symptoms [4].
MUAC status	MUAC >= 23.5*<br>MUAC < 23.5 cm	Maternal risk of malnutrition indicated by mid-upper arm circumference, with the cut-off points is 23.5 cm [5].
Residence location	Regency's capital*<br>Subdistricts closer to regency's capital<br>Subdistricts far from regency's capital	The location where the study participants lived.
Ethnicity	Indigenous (Banggai, Balantak, and Saluan tribes) *<br>Non-indigenous or immigrant	A particular ethnic group of the participants.
Smoking exposure	No*<br>Yes	The smoking status of either study participants or family members living in the same house as the study participants.
Father's education	Academy*<br>High school<br>Middle school<br>Primary school/ no school	Category of the paternal education level.
Number of children under five years	< Two children*<br>>= Two children	Category of the number of under-five children living in the house of the study participants.
Socioeconomic status (SES)	Mid-high SES*<br>Low SES	The composite measure of household's cumulative living of standard, which was formed using principal component analysis (PCA).[6] This variable was derived from 10 variables, including ownership of vehicles, the use of cooking oil, bathroom sewerage condition, kitchen sewerage condition, latrine facility, house's floor material, house's wall material, drink water source, clean water source and income.

An asterisk (*) is used to indicate the reference category for categorical predictor variables.

Table S3. Univariate logistic regression of associated factors of stunting in early and later infancy.

Associated factors	Early infancy		Later infancy	
	OR (95% CI) [a]	p	OR (95% CI) [a]	p
**Infant factors**				
Female	1.90 (0.83 – 4.37)	0.12†	1.33 (0.55 – 3.18)	0.52
Mixed or formula feeding	1.39 (0.37 – 5.18)	0.62	-	-
Not commenced complementary feeding at 6 months	-	-	0.68 (0.08 – 5.70)	0.72
Had morbidity	1.31 (0.52 – 3.29)	0.56	2.67 (1.07 – 6.67)	0.035*
Small-for-gestational-age	4.37 (1.68 – 11.31)	0.002*	7.49 (2.79 – 20.08)	<0.001
Short birth length	5.26 (1.95 – 14.17)	0.001*	3.17 (1.11 – 9.05)	0.03*
Caesarean section	0.60 (0.21 – 1.71)	0.33	0.55 (0.18 – 1.73)	0.31
Had pre-lacteal feeding	1.60 (0.70 – 3.64)	0.26	1.62 (0.67 – 3.89)	0.28
Non-early initiation of breastfeeding	1.40 (0.62 – 3.19)	0.42	0.78 (0.32 – 1.90)	0.59
**Maternal factors**				
Incomplete attendance for 4 ANC visits	0.47 (0.13 – 1.70)	0.25	0.16 (0.02 – 1.26)	0.08†
Preterm birth	5.92 (2.02 – 17.28)	0.001*	2.19 (0.69 – 6.95)	0.18†
Do not have family health insurance	2.48 (0.70 – 9.16)	0.17†	0.80 (0.16 – 3.96)	0.78
IG (Antenatal iron-folic acid)	2.13 (0.51 – 8.85)	0.30	1.00 (0.19 – 5.59)	1.00
Low supplements compliance	0.42 (0.10 – 1.93)	0.26†	0.88 (0.24 – 3.25)	0.84†
Maternal energy intake < RDA	1.79 (0.38 – 8.31)	0.45	3.28 (0.41 – 26.02)	0.26†
Maternal height <145 cm	1.37 (0.35 – 5.33)	0.65	2.64 (0.74 – 9.41)	0.13†
Maternal age at pregnancy at risk	0.69 (0.22 – 2.19)	0.53	1.20 (0.41 – 3.55)	0.74
Low mother's education	1.44 (0.46 – 4.54)	0.53	1.70 (0.47 – 6.15)	0.81
Maternal MUAC < 23.5 cm	2.84 (1.02 – 7.98)	0.04*	2.71 (0.92 – 7.98)	0.06†
**Household factors**				
Subdistricts far from regency's capital	2.91 (0.93 – 9.09)	0.06†	5.16 (1.53 – 17.41)	0.008*
Non-indigenous or immigrant	0.32 (0.13 – 0.74)	0.008*	0.36 (0.15 – 0.89)	0.02*
Smoking exposure	1.70 (0.67 – 4.33)	0.25	0.84 (0.34 – 2.08)	0.71
Number of children U2 in the house >2 children	0.80 (0.34 – 1.92)	0.62	0.69 (0.27 – 1.77)	0.43

OR = Odds Ratio, CI = Confidence Interval; [a] there is no multicollinearity between variables; ANC= Antenatal care, RDA= Recommended dietary allowance, MUAC= mid-upper arm circumference; *significant at the level <0.05; † variables were included in the multiple logistic regression model.

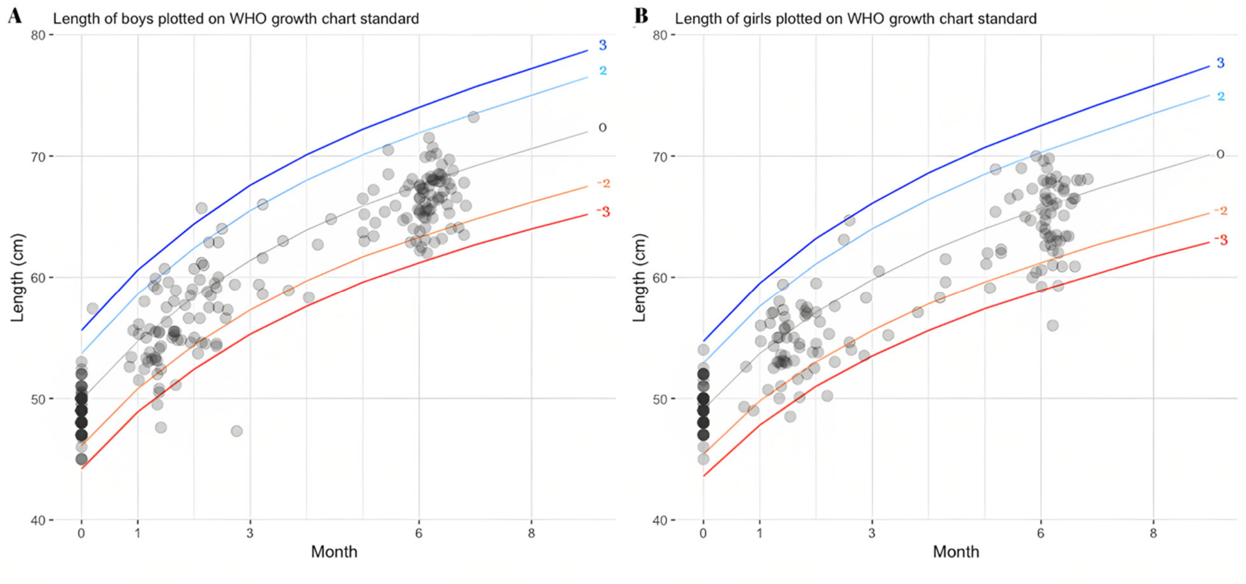

Figure S1. Length of boys (**A**) and girls (**B**) plotted on WHO growth chart.

1. WHO *Nutrition Landscape Information System (NLIS) country profile indicators: interpretation guide*; 2010; ISBN 9789415999555.
2. Pritchard, N.; Lindquist, A.; Siqueira, I. dos A.; Walker, S.P.; Permezel, M. INTERGROWTH-21st compared with GROW customized centiles in the detection of adverse perinatal outcomes at term. *J. Matern. Neonatal Med.* **2020**, *33*, 961–966, doi:10.1080/14767058.2018.1511696.
3. MoH *Laporan Nasional Riset Kesehatan Dasar (Riskesdas) tahun 2018*; Jakarta, Indonesia, 2019;
4. Westhuizen, C. van der; Wyatt, G.; Williams, J.K.; Stein, D.J.; Sorsdahl, K.; van der Westhuizen, C.; Wyatt, G.; Williams, J.K.; Stein, D.J.; Sorsdahl, K. Validation of the Self Reporting Questionnaire 20-Item (SRQ-20) for Use in a Low- and Middle-Income Country Emergency Centre Setting. *Int. J. Ment. Health Addict.* **2016**, *14*, 37–48, doi:10.1007/s11469-015-9566-x.
5. Sartika, A.N.; Khoirunnisa, M.; Meiyetriani, E.; Ermayani, E.; Pramesthi, I.L.; Nur Ananda, A.J. Prenatal and postnatal determinants of stunting at age 0–11 months: A cross-sectional study in Indonesia. *PLoS One* **2021**, *16*, 1–14, doi:10.1371/journal.pone.0254662.
6. Kumar, A.; Kumari, D.; Singh, A. Increasing socioeconomic inequality in childhood undernutrition in urban India: Trends between 1992-93, 1998-99 and 2005-06. *Health Policy Plan.* **2015**, *30*, 1003–1016, doi:10.1093/heapol/czu104.
